# The Silent Vehicle: Validating the Biological Signals and Noises of Baculovirus Vectors in Macrophage-Targeted Vaccines

**DOI:** 10.3390/vaccines14090816

**Published:** 2026-09-16

**Authors:** Beginda Ridwan, Farrah Putri Salmanida, Rika Wahyuningtyas, Yin-Siew Lai, Mei-Li Wu, Wen-Bin Chung, Chia-Tsai Chang, Ko-Tung Chang

**Affiliations:** 1 Department of Biological Science and Technology, National Pingtung University of Science and Technology, Neipu 912, Pingtung, Taiwan; kakavengeance77@gmail.com (B.R.); fapsa98@gmail.com (F.P.S.); rikawahyurizky@gmail.com (R.W.); rindy.0802@gmail.com (Y.-S.L.); 2 Department of Animal Product Technology, Faculty of Animal Science, Brawijaya University, Malang 65145, Indonesia; 3 Research Centre for Animal Biologics, National Pingtung University of Science and Technology, Neipu 912, Pingtung, Taiwan; mlwu@mail.npust.edu.tw (M.-L.W.); wbchung@mail.npust.edu.tw (W.-B.C.); 4 Department School of Life Sciences and Technology, Institut Teknologi Bandung, Bandung 40132, Indonesia; 5 Department of Food Science, National Pingtung University of Science and Technology, Neipu 912, Pingtung, Taiwan; 6 College of Veterinary Medicine, National Pingtung University of Science and Technology, Neipu 912, Pingtung, Taiwan; 7 School of Pharmacy, China Medical University, Taichung 406, Taiwan; kirbychang0524@gmail.com; 8 Center of Excellence for Metabolic Associated Fatty Liver Disease, National Sun Yat-Sen University, Kaohsiung 804, Taiwan

**Keywords:** TAMs, baculovirus, macrophages, recombinant protein, immunomodulation, M1, M2, tumor

## Abstract

Objective: The Baculovirus Expression Vector System (BEVS) is an established eukaryotic platform for high-yield recombinant protein production and is widely utilized for generating vaccine antigens, including recombinant classical swine fever virus E2 glycoprotein, African swine fever virus structural proteins (p72, p30, and p54), and porcine reproductive and respiratory syndrome virus envelope proteins GP5 and M. These antigens interact with macrophage-associated receptors, including CD163 and CD206, to modulate immune responses through macrophage polarization. However, whether baculovirus vector-derived components intrinsically influence macrophage polarization remains unclear, raising concerns regarding potential impacts on biological activity, vaccine efficacy, and downstream purification strategies. Methods: In this study, an M2-like tumor-associated macrophage (TAM) model capable of repolarizing toward an M1-like phenotype was established to distinguish the transcriptomic effects of the baculovirus vector control (VC) from those of the recombinant antigen (A1). Comparative RNA sequencing, Gene Set Enrichment Analysis (GSEA), functional enrichment analyses, and linear regression analysis were performed to evaluate vector- and antigen-associated transcriptional responses. Results: Comprehensive transcriptomic analysis showed that VC treatment induced only minimal transcriptomic alterations, with limited immune-related pathway enrichment and expression profiles that remained highly similar to those of untreated M2-like TAMs. Furthermore, linear regression analysis demonstrated negligible correlations between VC-induced and A1-induced transcriptional responses indicating that the limited changes associated with the vector backbone did not correspond to the receptor-mediated immune responses elicited by the recombinant antigen. In contrast, A1 stimulation induced distinct transcriptional reprogramming characterized by enrichment of pro-inflammatory and immune-activation pathways and a shift toward an M1-like macrophage phenotype. Conclusions: These findings suggest the negligible correspondence between VC- and A1-induced transcriptional profiles further supports the transcriptomic noises and signals of the baculovirus vector and inserted proteins, reinforcing its suitability as a platform for macrophage-targeted vaccine development while providing a basis for future optimization of downstream purification strategies.

## 1. Introduction

Macrophages are highly plastic innate immune cells that play essential roles in tissue homeostasis, pathogen clearance, antigen presentation, and regulation of adaptive immunity [1,2]. In response to environmental stimuli, macrophages can adopt diverse activation states ranging from classically activated pro-inflammatory phenotypes to alternatively activated anti-inflammatory phenotypes. This functional plasticity allows macrophages to orchestrate immune responses during infection, inflammation, and tumor progression [2,3]. Because of their central role in immune regulation, macrophages have emerged as attractive targets for vaccine development and immunotherapeutic interventions designed to enhance host-protective immunity [4,5].

Macrophages are particularly important in several economically significant swine viral diseases, including porcine reproductive and respiratory syndrome (PRRS), African swine fever (ASF), and classical swine fever (CSF). PRRSV preferentially infects porcine alveolar macrophages, while ASFV exhibits a strong tropism for cells of the monocyte–macrophage lineage, enabling these viruses to manipulate host immune responses and establish persistent infection [6,7]. Similarly, macrophage-associated immune dysregulation contributes substantially to the pathogenesis of CSF [8]. Consequently, vaccine strategies that effectively target macrophages have gained increasing attention as a means of improving antigen presentation, immune activation, and long-term protective responses against viral diseases [4,5].

Despite remarkable progress in recombinant vaccine technologies, the production of purified recombinant proteins often requires multiple expression, purification, and quality-control steps that increase manufacturing costs and limit large-scale implementation. These challenges are particularly relevant for veterinary vaccines, where economic feasibility is an important consideration. The Baculovirus Expression Vector System (BEVS) has emerged as one of the most successful recombinant protein-production platforms because it combines biosafety, scalability, and the ability to generate complex proteins with eukaryotic post-translational modifications [9,10]. Over the past three decades, BEVS has evolved from an experimental expression technology into a mainstream manufacturing platform used for the development of numerous commercial and experimental vaccines [10,11]. Licensed vaccine products produced using baculovirus-based technologies have further demonstrated the practical applicability and economic advantages of this platform for large-scale vaccine production [11].

Beyond recombinant protein production, baculoviruses possess intrinsic immunostimulatory properties that may contribute to vaccine-induced immunity. Previous studies demonstrated that baculoviral DNA can activate innate immune-signaling pathways through pattern-recognition receptors, resulting in the induction of type I interferons, inflammatory cytokines, and antiviral responses [12,13]. These observations have stimulated interest in baculovirus not only as an expression platform but also as a biologically active vaccine vector with potential adjuvant-like properties [11,14].

Our previous study [15] demonstrated that recombinant baculovirus constructs expressing PRRSV-associated antigens were capable of promoting macrophage repolarization from an M2-like phenotype toward a pro-inflammatory M1-like phenotype. This finding supports the concept of macrophage-targeted vaccination and suggests that baculovirus-based vaccines may actively influence macrophage functional plasticity. Because macrophage polarization is closely associated with the quality and magnitude of vaccine-induced immune responses, understanding the specific contribution of each vaccine component is essential for the rational design of next-generation vaccine platforms [3,5]. However, because previous baculovirus vaccine constructs contained both the recombinant antigen and the vector backbone, it remains unclear whether the observed macrophage modulation was driven by antigen expression, the baculovirus vector, or both. Therefore, this study investigated the transcriptomic influence of the baculovirus vector control (VC) in an in vitro-generated tumor-associated macrophage (TAM) model to determine whether the empty vector alone could induce transcriptional alterations associated with macrophage repolarization, thereby providing a basis for distinguishing vector-associated from antigen-specific immune responses.

## 2. Materials and Methods

### 2.1. Materials

RAW 264.7 cells (a mouse macrophage cell line) were obtained from the American Type Culture Collection (ATCC, Manassas, VA, USA) and 4T1 cells (a mouse breast cancer cell line) were purchased from Elabscience (Houston, TX, USA). For cell culture, Dulbecco’s modified Eagle’s medium (DMEM; Corning Inc., Corning, NY, USA), fetal bovine serum (FBS; HyClone Laboratories LLC, Logan, UT, USA), phosphate-buffered saline (PBS; J.T.Baker, Phillipsburg, NJ, USA), and trypan blue solution (Sigma-Aldrich, St. Louis, MO, USA) were used. The vector control (VC) was provided by the Research Center for Animal Biologics (RCAB), National Pingtung University of Science and Technology (NPUST), Pingtung, Taiwan.

### 2.2. Constructed the Recombinant Protein Antigen

The recombinant antigen was created by the Research Center of Animal Biologics (RCAB) at NPUST. A1 (g6Ld10T) was produced by combining the complete sequence of ORF5 with a portion of the ORF6 sequence, while also including T-cell epitopes. To facilitate the cloning and expression of the target protein, the recombinant antigens were deliberately designed to feature specific BamHI and EcoRI restriction sites. The recombinant antigen A1 was selected based on our previous study demonstrating its ability to repolarize M2-like macrophages toward an M1 phenotype and enhance anti-tumor immune responses [15]. Therefore, A1 served as an appropriate biological model to distinguish antigen-specific transcriptional responses from potential effects attributable to the baculovirus vector backbone.

### 2.3. Development of TAMs In Vitro

#### 2.3.1. Cell Culture

RAW264.7 murine macrophage cells were cultured in Dulbecco’s Modified Eagle Medium (DMEM) supplemented with 10% fetal bovine serum (FBS), while the 4T1 breast cancer cell line was maintained under identical conditions for the preparation of tumor-conditioned medium (TCM). Cells were incubated at 37 °C in a humidified atmosphere containing 5% CO_2_ and grown to approximately 70–80% confluence. For TCM preparation, the culture supernatant from 4T1 cells was collected, centrifuged to remove cellular debris, and subsequently filtered through a 0.22 µm syringe filter to ensure sterility prior to use. Six independent experimental runs were performed for each condition described in Section 2.4. In each experimental run, the phenotype of ivTAMs was assessed based on six macrophage-associated markers (IL-10, Arg-1, Caspase-6, CD206, TNF-α, and IL-1β), to confirm successful establishment of the ivTAM phenotype. Total RNA obtained from the six independent runs of each condition was subsequently pooled to generate one composite RNA-seq sample for NGS analyses.

#### 2.3.2. In Vitro Tumor-Associated Macrophages (ivTAMs)

Tumor-associated macrophages (TAMs) were generated in vitro by stimulating RAW264.7 cells with tumor-conditioned medium (TCM) derived from 4T1 breast cancer cells for 24 h. The use of tumor-conditioned medium to induce TAM-like macrophage polarization has been widely employed as an experimental model for investigating macrophage–tumor interactions and macrophage plasticity [4,16]. To prepare TCM, 4T1 cells were seeded at a density of approximately 1 × 10^6^ cells/mL and cultured in DMEM supplemented with 10% FBS for 24 h until reaching approximately 80% confluence. The culture supernatant was collected, centrifuged to remove cellular debris, and subsequently filtered through a 0.22 μm syringe filter prior to use for macrophage stimulation.

### 2.4. Recombinant Antigen Stimulation

Non-treated RAW264.7 cells and ivTAMs were used as negative controls. For stimulation experiments, recombinant antigen A1 (2 µg) was administered to ivTAMs. A baculovirus vector control (VC; 2 µg), consisting of the same baculovirus expression system without the inserted target gene, was included to account for any nonspecific effects derived from the viral vector itself. This control ensures that observed cellular responses are attributable to the recombinant antigen rather than baculovirus-mediated stimulation. Both A1 and VC treatments were applied to ivTAMs 24 h before sample collection. All treatments were applied under identical conditions for subsequent analysis. The purified baculovirus vector and antigen protein were supplied as a gift by the Research Center of Animal Biologics (RCAB, NPUST). The production of a recombinant protein in the baculovirus system is described in a previous study, including in the Supplementary Materials of the previous study [15].

### 2.5. RNA Isolation and Next-Generation Sequencing (NGS) Analysis

#### 2.5.1. RNA Extraction

Total RNA was extracted from cells using TRIzol reagent (Invitrogen, Waltham, MA, USA) according to the manufacturer’s instructions. The concentration and purity of the isolated RNA were determined prior to downstream applications.

#### 2.5.2. Next-Generation Sequencing (NGS) Analysis

RNA obtained from the six independent experimental runs corresponding to each condition was pooled prior to library preparation, generating one composite RNA-seq sample for each experimental condition. Thus, although six independent experimental runs were conducted, the RNA-seq dataset contained one pooled sequencing library per condition and did not retain replicate-level information. Consequently, biological dispersion and replicate-based confidence intervals could not be independently estimated from the sequencing dataset. Primary RNA-sequencing data processing and bioinformatic analysis were performed by Genomics BioSci & Tech Co. (New Taipei City, Taiwan) according to the pipeline documented in the company’s analysis report. Raw sequencing reads were quality-filtered using Trimmomatic (v0.36) [17], followed by alignment to the reference genome using Bowtie2 (v2.3.5) [18]. Gene expression levels were quantified using RSEM (v1.3.3) [19], and differential gene expression analysis was performed using the edgeR package (v3.16.5) [20]. Sequencing libraries were quality-assessed using an Agilent 2100 BioAnalyzer (Agilent Technologies, Santa Clara, CA, USA) and quantified by real-time PCR prior to sequencing on an Illumina NovaSeq 6000 platform (Illumina, Inc., San Diego, CA, USA) to generate 150-bp paired-end reads. Ribosomal RNA sequences were removed before alignment of the filtered reads to the reference genome for differential expression analysis. The software versions and bioinformatic procedures described above correspond to those specified in the original analysis report provided by the sequencing company. Differential gene-expression outputs were generated by the sequencing company’s original bioinformatic pipeline using edgeR, with |Log2FC| > 1 and an adjusted *p*-value (q-value) < 0.05 applied as the original filtering criteria. Because sequencing was performed on one pooled library per condition, the resulting statistical values do not represent replicate-based estimates of biological variability. Accordingly, the present study primarily uses the RNA-seq data for descriptive and exploratory transcriptomic comparisons. The RNA sequencing data generated in this study have been deposited in the NCBI BioProject database under accession number PRJNA1032217. The datasets presented in this study can be found in online repositories (NCBI Bioproject PRJNA1032217: https://www.ncbi.nlm.nih.gov/bioproject/PRJNA1032217) (accessed on 13 August 2026).

### 2.6. Transcriptome Analysis

#### 2.6.1. Over-Representation Analysis (ORA)

For exploratory transcriptomic analysis, genes were filtered using |log2 fold change (log2FC)| > 1 together with the q-value < 0.05 reported by the original sequencing company pipeline. Because replicate-level sequencing information was not available following sample pooling, these criteria were used for descriptive gene prioritization rather than replicate-based statistical inference. Volcano plots were generated using SRplot [21] to visualize the distribution of DEGs. Venn diagram analysis was performed to compare gene overlaps between experimental groups. Over-representation analysis (ORA) was conducted separately for upregulated and downregulated DEGs to identify significantly enriched functional categories. Gene Ontology (GO) and Kyoto Encyclopedia of Genes and Genomes (KEGG) pathway-enrichment analyses were performed using the ShinyGO web-based platform [22]. A custom background gene set consisting of expressed genes was applied to improve enrichment accuracy. Statistical significance was determined using an FDR threshold of <0.05, with gene sets filtered by a minimum size of 50 and a maximum size of 5000 genes [23,24].

#### 2.6.2. Gene Set Enrichment Analysis (GSEA)

Gene Set Enrichment Analysis (GSEA) was performed using GSEA software (v4.3.2, Broad Institute) to identify coordinated changes in predefined gene sets. A pre-ranked gene list was generated by ordering all detected protein-coding genes based on the signal-to-noise ratio derived from normalized expression values between experimental and control groups. Enrichment analysis was conducted against the Molecular Signatures Database (MSigDB v7.5), including Hallmark (H), KEGG (C2: CP), and Gene Ontology Biological Process (C5: BP) gene sets [25,26]. Enrichment scores were calculated using 1000 permutations, and gene sets with fewer than 15 or more than 500 genes were excluded. Significantly enriched pathways were defined by an FDR q-value < 0.25 and an absolute normalized enrichment score (|NES|) > 1.5. Leading-edge analysis was performed to identify core genes contributing to the enrichment signal.

## 3. Results

### 3.1. Transcriptomic Profiling Confirms Successful Establishment of In Vitro TAM-like Macrophages and Reveals Minimal Influence of Baculovirus Vector Control

We had established ivTAMs in our lab previously [27] according to the study of Yao et al. [28]. Prior to transcriptomic analysis, the phenotype of ivTAMs was preliminarily confirmed by quantitative real-time PCR (RT-qPCR) and the result is summarized in Table 1. Across the six examined markers (*IL-10*, *Arg-1*, *Caspase-6*, *CD206*, *TNF-α*, and *IL-1β*), the overall expression pattern of ivTAMs in our lab corresponded to that reported in the reference TAM model. The concordant expression patterns of the ivTAMs between two labs provided the foundation that has been laid for the following NGS analysis. 

Following comprehensive phenotype confirmation of ivTAMs, transcriptomic analyses were performed to evaluate the influence of the baculovirus vector control (VC) on the macrophage. According to our data, alignment of the same pathways in the ivTAMs versus ivTAMs + VC comparison showed little or no detectable enrichment, suggesting comparable pathway activity between the two groups. The absence of substantial pathway shifts suggests that VC treatment induced only limited transcriptomic alterations in the immune-related transcriptional programs established during TAM induction. Collectively, these findings indicate that the transcriptomic characteristics distinguishing TAM-like macrophages from naïve RAW264.7 cells were largely preserved following VC treatment under the experimental conditions evaluated. 

**Figure 1:**
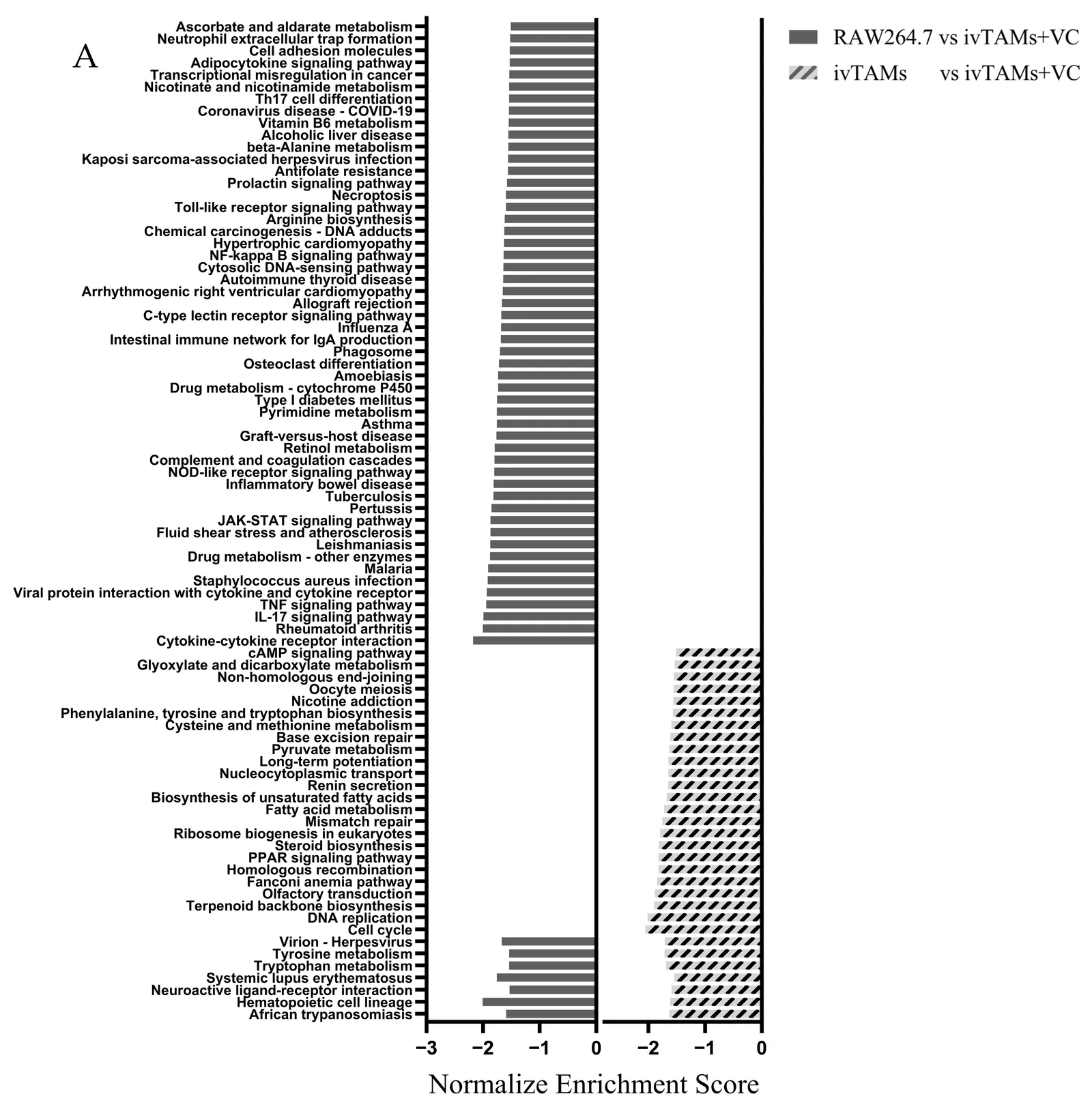

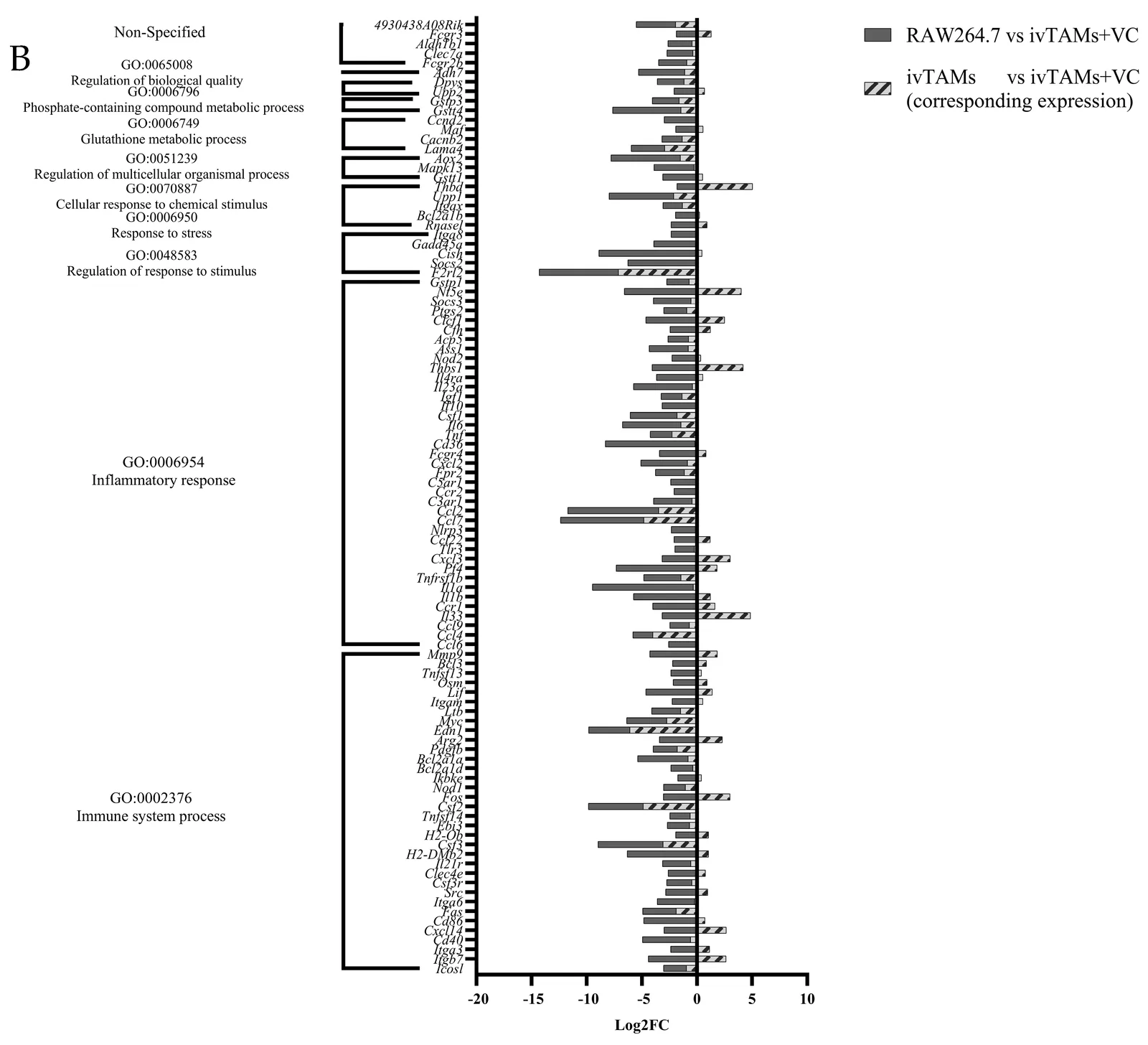
(**A**) Downregulated Gene Set Enrichment Analysis (GSEA) comparing RAW264.7 vs. ivTAMs + VC (solid gray) and ivTAMs vs. ivTAMs + VC (s gray hatching). Enrichment is presented as normalized enrichment score (NES). Immune- and inflammation-related pathways, including cytokine–cytokine receptor interaction, TNF signaling, NF-κB signaling, Toll-like receptor signaling and JAK–STAT signaling were predominantly enriched in the RAW264.7 comparison, whereas metabolic- and proliferation-associated pathways, such as DNA replication, steroid biosynthesis, and fatty acid metabolism, were enriched in the ivTAMs comparison. Pathways without visible enrichment in the aligned comparison indicate relatively comparable pathway activity between groups, whereas pathways displaying NES values represent relatively lower expression or enrichment in ivTAMs + VC compared to the corresponding control. Therefore, during pathway alignment analysis, pathways lacking visible NES enrichment were interpreted as having relatively higher transcriptional activity than pathways exhibiting negative NES values, enabling discrimination between conserved and suppressed biological processes associated with TAM polarization. (**B**) Gene-level validation of TAM-associated transcriptional features. Top 100 differentially expressed genes were filtered based on pathway overlap (genes present in ≥2 enriched pathways) and expression magnitude (Log2 fold change < −1.0). The expression pattern shows the difference between RAW264.7 and ivTAMs.

To further validate TAM-associated transcriptional signatures, genes were filtered using three criteria: (i) occurrence in at least two enriched pathways, (ii) pathway association identified through GSEA, and (iii) differential expression with Log2 fold change < −1.0. Gene-level analysis revealed consistent upregulation of multiple immune- and inflammation-associated genes in ivTAMs + VC relative to RAW264.7 cells, including cytokines (*Il6*, *Tnf*), chemokines (*Ccl2*, *Ccl4*, *Ccl6*, *Cxcl2*, *Cxcl3*), pattern-recognition receptors (*Nod2*, *Nlrp3*), antigen-presentation molecules (*H2-DMb2*, *H2-Ob*), and inflammatory mediators (*Ptgs2*, *Socs3*) (Figure 1B). Importantly, these genes exhibited substantially smaller expression differences in the ivTAMs versus ivTAMs + VC comparison, indicating that their expression levels were largely preserved following VC treatment. Together, these results support successful generation of TAM-like macrophages in vitro and suggest that the baculovirus vector control was associated with comparatively limited transcriptomic perturbations under the experimental conditions evaluated.

### 3.2. Cross-Species Comparison Reveals Conserved Transcriptional Signatures Between Murine ivTAMs and Human Breast Cancer TAMs

**Figure 2:**
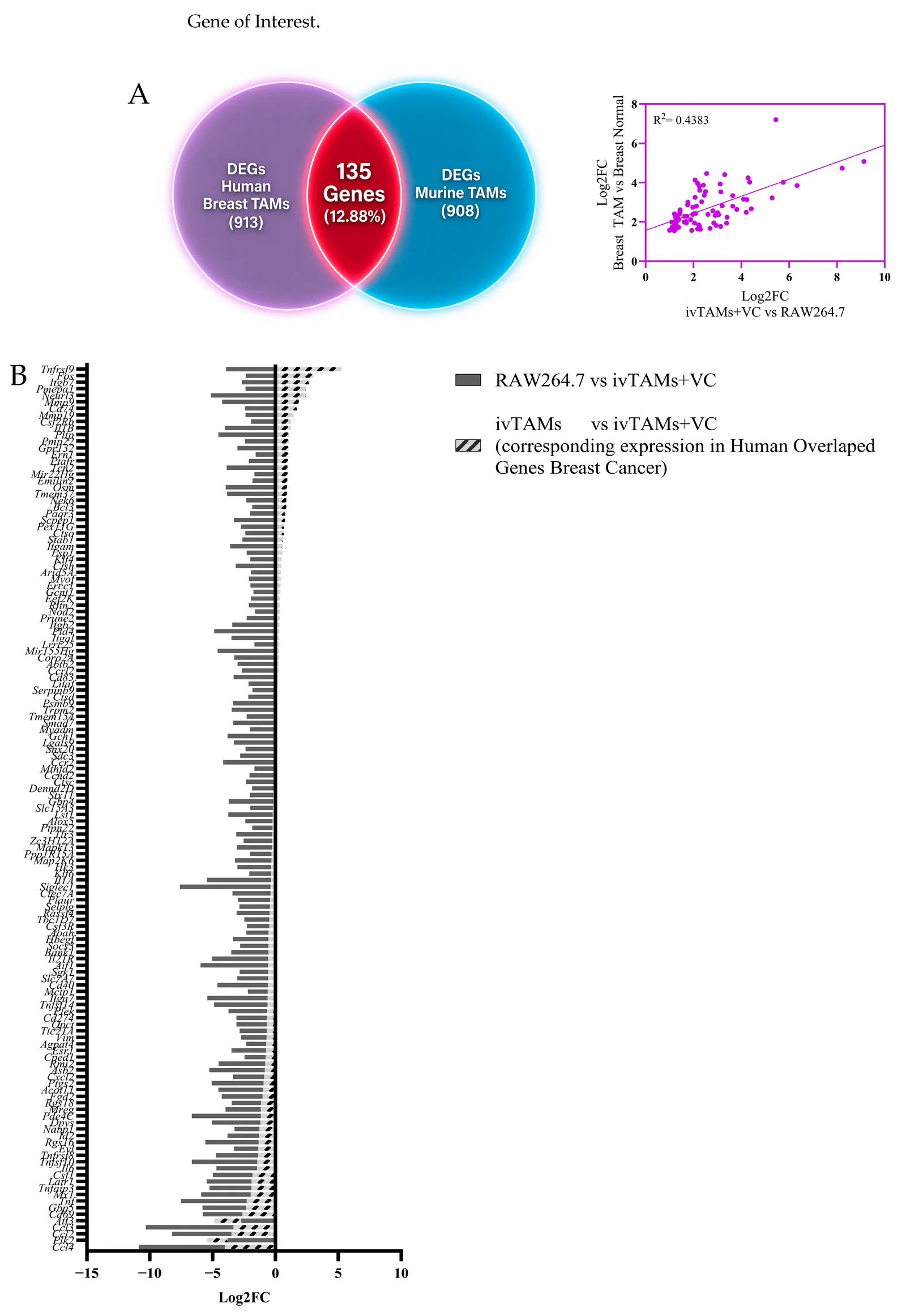
(**A**) Venn diagram showing overlap between differentially expressed genes (DEGs) derived from published human breast cancer TAM datasets and DEGs identified from the murine ivTAMs + VC vs. RAW264.7 comparison. A total of 135 overlapping genes were identified, representing conserved TAM-associated transcriptional signatures shared between human and murine datasets. Among the 135 overlapping genes, 88 genes exhibited less than a two-fold difference in relative expression changes between the human and murine datasets, corresponding conceptually to less than one cycle difference in PCR-based amplification. Regression analysis of these 88 genes yielded an R^2^ value of 0.4383, indicating a positive correspondence in their expression patterns across the two datasets. (**B**) Expression profiling of the 135 overlapping genes identified between human and murine TAM datasets. Bar plots represent Log2 fold change values from the RAW264.7 vs. ivTAMs + VC comparison (gray bars), while striped bars indicate corresponding expression patterns associated with overlapping human breast cancer TAM genes. Transcripts detected in both human and murine TAM datasets, whereas black-labeled genes represent transcripts derived exclusively from the human TAM-associated dataset. The overall expression pattern demonstrates conservation of TAM-associated transcriptional programs between species and supports the biological relevance of the in vitro-generated murine TAM model.

To evaluate the biological relevance of the in vitro-generated TAM model, differentially expressed genes (DEGs) identified in the RAW264.7 versus ivTAMs + VC comparison were compared with a published human breast cancer TAM transcriptomic dataset (PMCID: PMC6472943). Cross-species comparison identified 135 overlapping genes shared between human breast cancer TAMs and murine ivTAMs, representing 12.88% of the human TAM-associated gene set (Figure 2A). In contrast, 913 genes were uniquely detected in the human dataset, whereas 908 genes were specific to the murine dataset. The 12.88% overlap indicates partial conservation of TAM-associated transcriptional features between the human breast cancer dataset and the murine ivTAM model, while the larger non-overlapping fractions highlight substantial species-, model-, and context-specific transcriptional differences.

To further characterize these conserved signatures, expression patterns of the 135 overlapping genes were examined in the murine ivTAMs + VC model (Figure 2B). According to our data, these 135 genes were more expressed in ivTAMs compared to RAW264.7, consistent with the result of GSEA in Figure 1. Notably, among the TAM-related genes conserved between human and mice, 23 genes were also appeared from the GSEA results (Table 2), highlighting the important roles played by these genes in TAMs.

Notably, expression levels of the overlapping genes remained largely unchanged in the ivTAMs versus ivTAMs + VC comparison, suggesting that the conserved TAM-associated transcriptional program was maintained following VC treatment. Collectively, these findings demonstrate that the in vitro-generated murine TAM model shares a subset of TAM-associated transcriptional characteristics observed in human breast cancer TAMs and further support the minimal influence of the baculovirus vector control on established TAM-associated gene-expression patterns.

To determine the functional behavior of these genes, we analyzed their expression patterns across two comparative conditions: ivTAMs + VC vs. RAW264.7 and ivTAMs + VC vs. ivTAMs + A1. In the first comparison, the selected genes exhibited consistently elevated expression levels in ivTAMs + VC relative to RAW264.7 cells, confirming their association with TAM-like polarization. In contrast, the same gene set showed markedly reduced expression in the presence of A1 (ivTAMs + VC vs. ivTAMs + A1), as demonstrated by the line plot analysis. This inverse expression pattern indicates that while these genes are characteristic of TAM-associated and breast cancer-related macrophage signatures, their expression is suppressed upon A1 treatment. Collectively, these results demonstrate that A1 modulates a conserved TAM-associated transcriptional program linked to breast cancer, suggesting a functional shift away from a protumor macrophage phenotype.

### 3.3. Classification of Conserved TAM-Associated Genes Reveals Limited Transcriptional Influence of VC and Distinct Responses Following A1 Treatment

To facilitate biological interpretation of the transcriptomic data, genes were categorized using a commonly adopted RNA-sequencing threshold based on Log2 fold change (Log2FC). A cutoff of Log2FC >1 or <−1, corresponding to an approximately two-fold change in gene expression, was selected because it is widely used to identify biologically meaningful transcriptional alterations while minimizing background variation. Genes with Log2FC values between −1 and 1 were considered relatively stable and were used to represent transcripts exhibiting minimal response to VC treatment. This classification was not intended to define absolute biological states but rather to provide an analytical framework for distinguishing VC-responsive genes from genes with limited detectable responses to VC and to facilitate subsequent comparison of antigen (A1)-mediated transcriptional responses.

**Figure 3:**
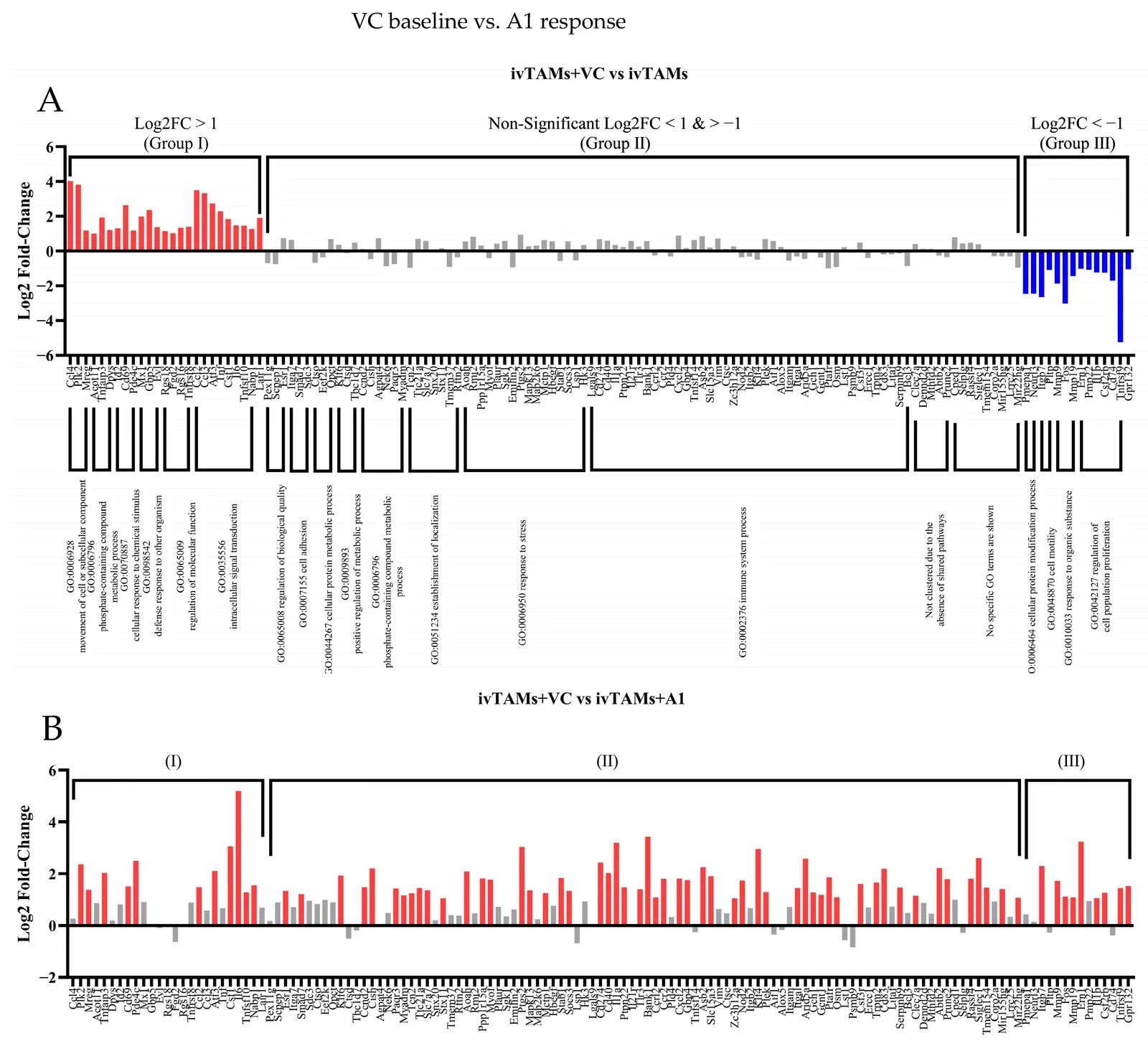
(**A**) The 135 conserved TAM-associated genes were classified according to Log2 fold-change values obtained from the ivTAMs + VC versus ivTAMs comparison. Red bars indicate significantly upregulated genes (Log2FC > 1), gray bars indicate genes with non-significant/minimal expression changes (−1 < Log2FC < 1), and blue bars indicate significantly downregulated genes (Log2FC < −1). Genes were categorized into Group I (Log2FC > 1), representing genes transcriptionally influenced by VC; Group II (−1 < Log2FC < 1), representing genes exhibiting minimal transcriptional responses to VC; and Group III (Log2FC < −1), representing genes whose expression was reduced following VC treatment. Major Gene Ontology (GO) biological functions associated with each gene cluster are indicated below the corresponding gene groups. This classification was subsequently used as the reference framework for evaluating antigen-specific transcriptional responses. (**B**) Expression profiles of the identical 135 conserved TAM-associated genes in the ivTAMs + VC versus ivTAMs + A1 comparison while preserving the Group I–III classification established in panel (**A**). Red, gray, and blue bars correspond to significantly upregulated, non-significantly changed, and significantly downregulated genes, respectively, according to the same Log2FC thresholds defined in panel (**A**). This analysis illustrates the differential transcriptional responses induced by recombinant A1 within VC-responsive genes (Group I), genes with limited detectable responses to VC (Group II), and VC-suppressed (Group III) gene subsets, thereby distinguishing antigen-specific biological activity from transcriptional changes attributable to the baculovirus vector backbone.

To systematically distinguish transcriptional changes attributable to the baculovirus vector control (VC) from those induced by the recombinant antigen, the 135 conserved TAM-associated genes identified from the human–murine overlap analysis were classified according to their expression changes in the ivTAMs + VC versus ivTAMs comparison (Figure 3A). Genes with Log2FC > 1, −1 < Log2FC < 1, and Log2FC < −1 were assigned to Groups I, II, and III, respectively. Although most genes showed minimal changes following VC treatment (Group II), a smaller subset, particularly Group III, exhibited marked downregulation, indicating that VC could partially modulate specific components of the ivTAM transcriptional profile. This classification therefore allowed VC-associated changes to be distinguished from subsequent A1-mediated responses.

Group I consisted of genes that were modestly induced by VC treatment. Functional annotation indicated that these genes were primarily associated with intracellular signal transduction, cellular movement, regulation of molecular function, defense response, and cellular responses to chemical stimuli. Because these genes were already activated by VC alone, subsequent transcriptional changes following A1 stimulation could not be unequivocally attributed to the recombinant antigen. Therefore, Group I was regarded as representing vector-associated background transcription (noise).

In contrast, the majority of conserved TAM-associated genes were distributed within Group II, where expression remained between Log2FC −1 and 1 following VC treatment. Functional annotation demonstrated enrichment of immune system processes, immune response, response to stress, intracellular signaling, regulation of cell activation, cellular localization, and multiple immune-regulatory pathways. The minimal transcriptional alteration observed in this group indicated that these biological programs were largely unaffected by the baculovirus vector itself. Consequently, genes within Group II provided the most appropriate background for evaluating antigen-specific biological activity (signals).

Group III comprised genes exhibiting marked negative Log2FC values following VC treatment, representing transcripts whose expression decreased after vector exposure. Functional annotation showed enrichment of cell population proliferation, protein modification, regulation of proliferation, cellular motility, and additional immune-associated biological processes. Because these genes were already substantially reduced following VC treatment, they provided a complementary dataset for determining whether A1 could further suppress pathways already influenced by the vector.

To evaluate antigen-specific activity, the identical gene order and Group I–III classification established in Figure 3A were retained and reanalyzed using the ivTAMs + VC versus ivTAMs + A1 comparison (Figure 3B). Distinct transcriptional behaviors were observed among the three predefined groups. Genes within Group I generally exhibited higher positive Log2FC values following A1 treatment; however, because these genes had already been altered by VC, these responses were interpreted primarily as amplification of vector-responsive transcription rather than definitive antigen-specific regulation.

Conversely, widespread transcriptional alterations were observed throughout Group II. Numerous genes that remained essentially unchanged following VC treatment displayed pronounced expression changes after A1 stimulation, demonstrating that the recombinant antigen effectively regulated transcription independently of vector-derived effects. These findings indicate that the biological activities represented by Group II predominantly reflect A1-specific immune modulation rather than residual effects of the baculovirus backbone. In other words, conserved genes in TAMs were actually downregulated by A1 stimulation, reflecting M2-like-to-M1 repolarization.

A similar pattern was observed in Group III. Although these genes had already exhibited decreased expression following VC treatment, A1 produced substantially larger positive Log2FC values in the ivTAMs + VC versus ivTAMs + A1 comparison. Because positive Log2FC in this comparison indicates lower transcript abundance in A1-treated cells relative to VC-treated cells, these results demonstrate that A1 further suppressed the expression of genes already reduced by VC. Collectively, these findings indicate that the recombinant antigen retained its capacity to regulate both Group II genes exhibiting limited detectable responses to VC (Group II) and VC-responsive genes (Group III), whereas transcriptional responses within Group I were largely attributable to vector-associated background activity.

### 3.4. Comparative Regression and Principal Component Analyses Reveal Distinct Transcriptional Patterns Following VC and A1 Treatment

To investigate whether the transcriptional responses induced by A1 were associated with expression changes previously observed following VC treatment, linear regression analyses were performed separately for Groups I, II, and III. Log2FC values obtained from the ivTAMs + VC versus ivTAMs comparison were used as the independent variable, whereas corresponding Log2FC values from the ivTAMs + VC versus ivTAMs + A1 comparison were used as the dependent variable.

**Figure 4:**
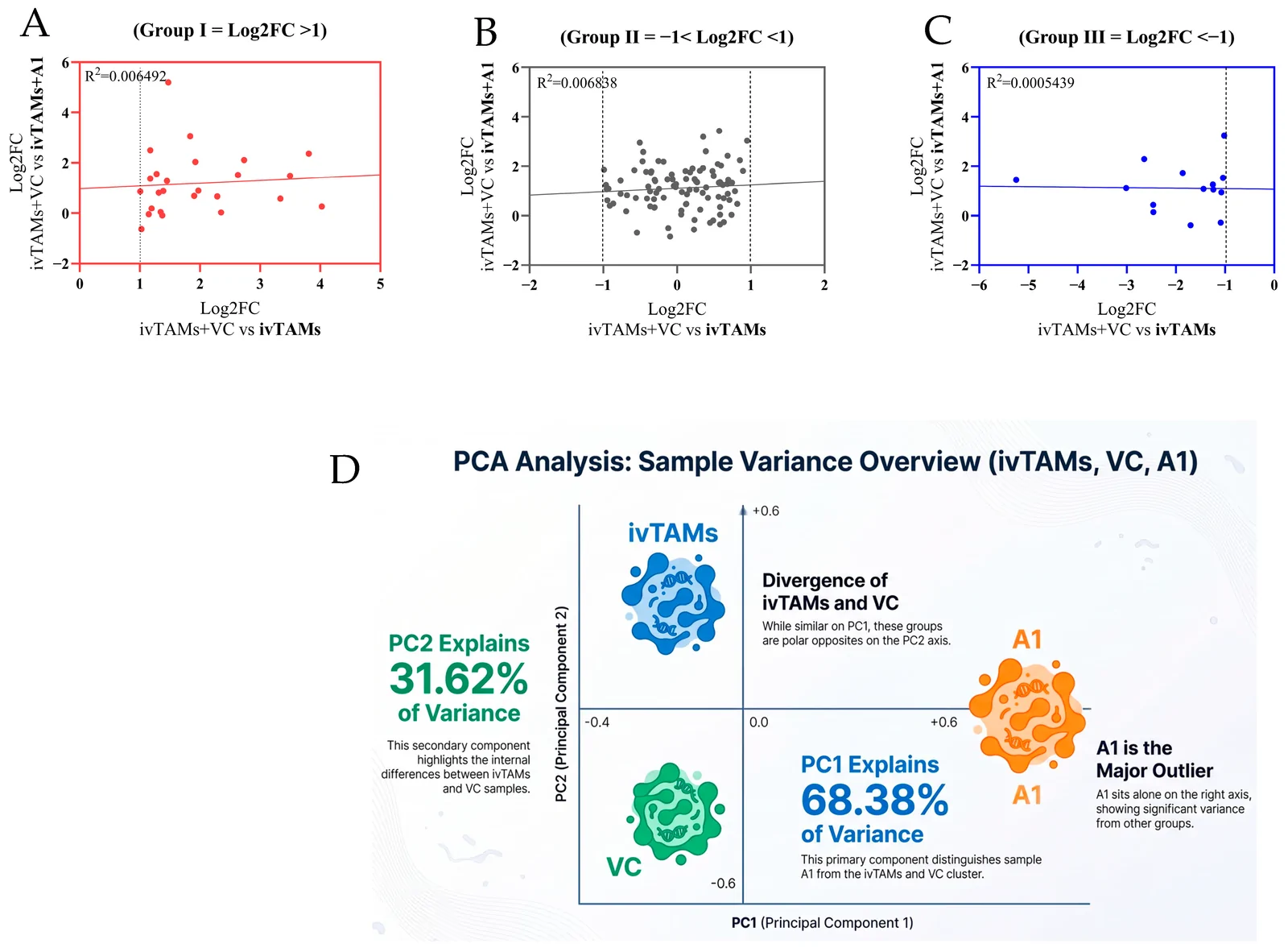
(**A**) Linear regression analysis of Group I genes (Log2FC > 1). Genes classified as Group I in the ivTAMs + VC versus ivTAMs comparison were subjected to linear regression analysis using corresponding Log2FC values from the ivTAMs + VC versus ivTAMs + A1 comparison. The *x*-axis represents transcriptional changes induced by VC treatment, whereas the *y*-axis represents changes induced by A1 stimulation. The coefficient of determination (R^2^) was calculated to evaluate the relationship between VC-associated and A1-associated transcriptional responses among genes positively regulated following VC treatment. (**B**) Linear regression analysis of Group II genes (−1 < Log2FC < 1). Genes exhibiting relatively stable expression following VC treatment were analyzed by comparing their corresponding Log2FC values in the ivTAMs + VC versus ivTAMs and ivTAMs + VC versus ivTAMs + A1 comparisons. Dashed vertical lines indicate the Group II classification boundaries In panels (**A**–**C**), dashed vertical lines indicate the predefined Log2FC classification boundaries used to define Groups I–III (Group I, Log2FC > 1; Group II, −1 < Log2FC < 1; Group III, Log2FC < −1). Linear regression analysis was performed to assess the degree of similarity between VC-induced and A1-induced transcriptional responses within the stable gene population. (**C**) Linear regression analysis of Group III genes (Log2FC < −1). Genes classified as strongly downregulated following VC treatment were evaluated by comparing their corresponding expression values between the ivTAMs + VC versus ivTAMs and ivTAMs + VC versus ivTAMs + A1 comparisons. The coefficient of determination (R^2^) was used to determine whether A1-mediated transcriptional responses were associated with the expression patterns previously observed following VC treatment. (**D**) Exploratory principal component analysis (PCA) of the pooled ivTAMs, ivTAMs + VC, and ivTAMs + A1 transcriptomic profiles. PC1 and PC2 accounted for 68.38% and 31.62% of the total variance, respectively. Each point represents one pooled RNA-seq library corresponding to an experimental condition. PCA was used to visualize relative global transcriptional relationships and was not interpreted as replicate-based statistical evidence of group separation. The PCA was generated in Galaxy and visually reformatted using Google NotebookLM.

For Group I genes (Log2FC > 1), regression analysis demonstrated an extremely weak relationship between VC-associated and A1-associated expression patterns, with an R^2^ value of 0.0065 (Figure 4A). Similarly, Group II genes (−1 < Log2FC < 1) exhibited minimal correlation between the two comparisons (R^2^ = 0.0068; Figure 4B). The weakest relationship was observed in Group III genes (Log2FC < −1), which displayed an R^2^ value of only 0.0005 (Figure 4C). 

Despite being classified according to their response to VC treatment, genes from all three groups exhibited little linear correspondence between their VC-associated and A1-associated log2FC patterns. These low R^2^ values indicate limited linear correspondence between the two transcriptional response patterns within the analyzed gene sets. However, because the RNA-seq samples were pooled prior to sequencing, the regression analysis cannot distinguish biological divergence from variability that would otherwise be quantified using independently sequenced biological replicates. Therefore, the regression analysis is interpreted descriptively and is not considered evidence of biological or mechanistic independence.

Exploratory PCA further demonstrated distinct global transcriptional positioning among ivTAMs, VC, and A1 (Figure 4D). PC1 accounted for 68.38% of the total variance and positioned ivTAMs and VC relatively close to each other, whereas A1 was clearly separated along this principal component. In contrast, PC2, accounting for 31.62% of the variance, distinguished VC from ivTAMs, indicating that VC treatment was associated with detectable transcriptional variation. Collectively, the PCA suggests that although VC was not transcriptionally identical to untreated ivTAMs, the dominant variation represented by PC1 distinguished A1 from both ivTAMs and VC. Because each condition was represented by a pooled RNA-seq sample, this PCA was interpreted as an exploratory visualization of transcriptomic relationships rather than statistical evidence of between-group separation.

### 3.5. Functional Enrichment Analysis of Group I Genes Reveals Immune-Associated Transcriptional Programs

**Figure 5:**
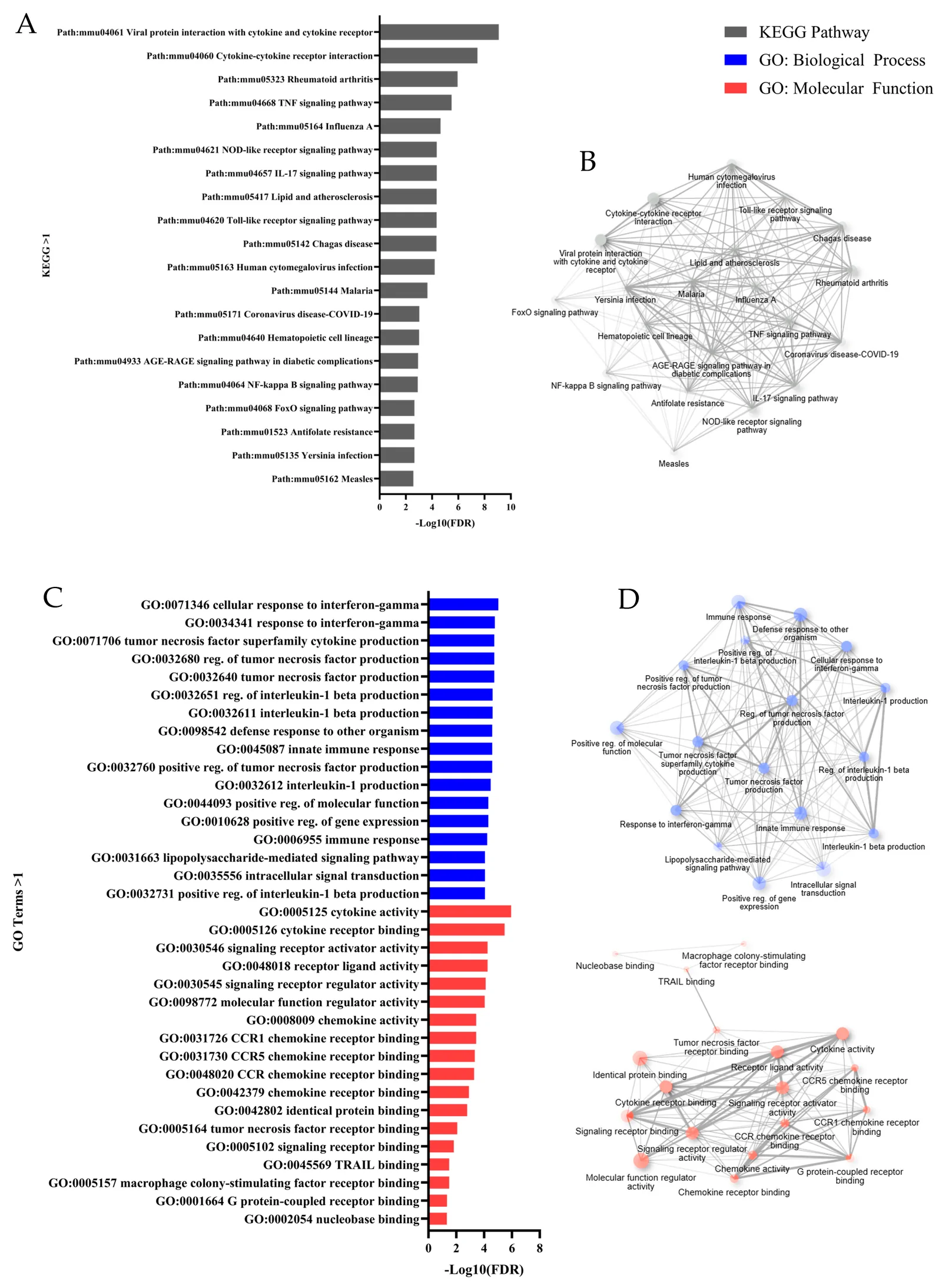
(**A**) KEGG pathway-enrichment analysis of Group I genes (Log2FC > 1). Differentially expressed genes classified within Group I were subjected to KEGG pathway-enrichment analysis. Significantly enriched pathways are presented according to −Log10(FDR). Enriched pathways were predominantly associated with cytokine signaling, innate immune responses, inflammatory signaling, and host–pathogen interactions. (**B**) Functional interaction network of enriched KEGG pathways identified in Group I genes. Network analysis was performed using significantly enriched KEGG pathways. Nodes represent enriched pathways, whereas connecting edges indicate shared genes between pathways. Pathways with greater connectivity represent highly interconnected biological processes involved in immune regulation, inflammatory responses, and cytokine-mediated signaling. (**C**) Gene Ontology (GO) enrichment analysis of Group I genes. Significantly enriched GO terms were identified from genes classified within Group I. Blue bars represent Biological Process (BP) categories, whereas red bars represent Molecular Function (MF) categories. Enriched biological processes were mainly associated with interferon-γ responses, tumor necrosis factor production, innate immune responses, cytokine signaling, and intracellular signal transduction. Enriched molecular functions were primarily related to cytokine activity, cytokine-receptor binding, chemokine activity, receptor ligand activity, and signaling receptor regulation. (**D**) Functional interaction network of enriched molecular functions identified in Group I genes. Nodes represent significantly enriched molecular functions, while edges indicate shared genes among GO terms. Highly connected clusters were primarily associated with cytokine activity, cytokine-receptor binding, chemokine-receptor binding, receptor ligand activity, and signaling receptor regulation, highlighting extensive functional interactions among immune-regulatory genes.

To investigate the biological functions represented by genes classified within Group I (Log2FC > 1), KEGG and Gene Ontology enrichment analyses were performed. KEGG pathway analysis revealed significant enrichment of immune- and inflammation-associated pathways, including viral protein interaction with cytokines and cytokine receptors, cytokine–cytokine receptor interaction, TNF signaling pathways, NOD-like receptor signaling pathways, IL-17 signaling pathways, Toll-like receptor signaling pathways, NF-κB signaling pathways, and hematopoietic cell lineage (Figure 5A). Several infection-related pathways, including Influenza A, Human cytomegalovirus infection, Coronavirus disease (COVID-19), Yersinia infection, Malaria, and Chagas disease, were also enriched due to extensive overlap of host immune-response genes.

Network analysis demonstrated strong interconnectivity among the enriched pathways (Figure 5B). Cytokine–cytokine receptor interaction, TNF signaling, IL-17 signaling, NOD-like receptor signaling, and Toll-like receptor signaling occupied central positions within the network, indicating extensive gene sharing among these pathways.

GO enrichment analysis further supported the predominance of immune-associated biological functions (Figure 5C). The most significantly enriched biological processes included cellular response to interferon-γ, regulation of tumor necrosis factor production, interleukin-1β production, innate immune response, immune response, lipopolysaccharide-mediated signaling pathway, and intracellular signal transduction. Molecular function enrichment identified cytokine activity, cytokine-receptor binding, chemokine activity, receptor ligand activity, signaling receptor activator activity, and chemokine-receptor binding as the dominant functional categories.

Consistent with these findings, molecular function network analysis demonstrated extensive connectivity among cytokine- and chemokine-related activities (Figure 5D). Cytokine activity, cytokine-receptor binding, chemokine-receptor binding, and receptor ligand activity formed the major functional hubs within the network.

### 3.6. Functional Characterization of Group II Genes Identifies Conserved TAM-Associated Pathways Minimally Affected by VC

**Figure 6:**
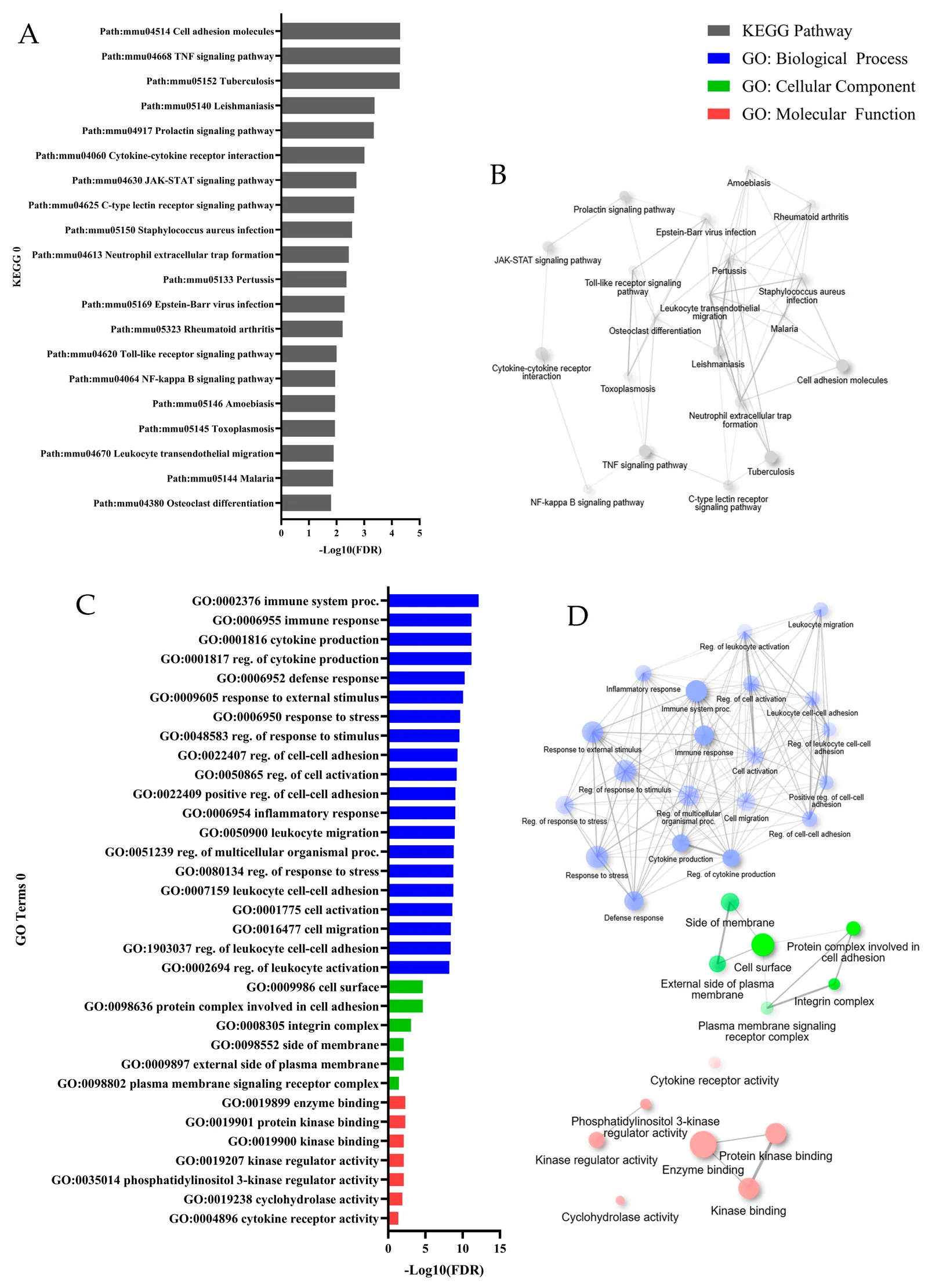
(**A**) KEGG pathway-enrichment analysis of Group II genes (−1 < Log2FC < 1). Conserved TAM-associated genes exhibiting minimal transcriptional changes following VC treatment were subjected to KEGG enrichment analysis. Significantly enriched pathways are presented according to −Log10(FDR). Enriched pathways were predominantly associated with immune regulation, cytokine signaling, leukocyte migration, cell adhesion, inflammatory responses, and host-defense mechanisms. (**B**) Functional interaction network of enriched KEGG pathways identified in Group II genes. Nodes represent significantly enriched KEGG pathways, while connecting edges indicate shared genes among pathways. Highly interconnected clusters were primarily associated with cytokine-mediated signaling, TNF signaling, JAK–STAT signaling, Toll-like receptor signaling, leukocyte migration, and cell-adhesion processes. (**C**) Gene Ontology enrichment analysis of Group II genes. Biological Process (BP), Cellular Component (CC), and Molecular Function (MF) enrichment analyses were performed using genes classified within Group II. Significantly enriched BP terms included immune response, cytokine production, inflammatory response, leukocyte migration, cell activation, and regulation of response to external stimuli. Enriched CC terms were mainly associated with cell surface structures, plasma membrane signaling receptor complexes, and integrin-containing complexes. Enriched MF terms included enzyme binding, kinase binding, protein kinase binding, kinase-regulator activity, phosphatidylinositol 3-kinase-regulator activity, and cytokine-receptor activity. (**D**) Functional interaction networks of enriched GO Biological Process (BP), Cellular Component (CC), and Molecular Function (MF) categories identified in Group II genes. Nodes represent enriched GO terms, while edges indicate shared genes among functional categories. The networks reveal extensive connectivity among immune-regulatory processes, membrane-associated signaling structures, and kinase-mediated signaling activities.

To determine the biological functions represented by genes exhibiting minimal responsiveness to VC treatment, enrichment analyses were performed using genes classified within Group II (−1 < Log2FC < 1). KEGG analysis revealed significant enrichment of immune-related pathways, including TNF signaling, cytokine–cytokine receptor interaction, JAK–STAT signaling, Toll-like receptor signaling, C-type lectin receptor signaling, leukocyte transendothelial migration, cell adhesion molecules, neutrophil extracellular trap formation, and NF-κB signaling (Figure 6A). Multiple infection-associated pathways, including Tuberculosis, Leishmaniasis, Staphylococcus aureus infection, Pertussis, Epstein–Barr virus infection, Toxoplasmosis, and Malaria, were also significantly enriched.

KEGG network analysis demonstrated extensive pathway interconnectivity (Figure 6B). TNF signaling, Toll-like receptor signaling, cytokine–cytokine receptor interaction, JAK–STAT signaling, and cell adhesion molecules formed major interaction hubs, suggesting that these pathways share substantial numbers of immune-regulatory genes.

GO enrichment analysis further supported the predominance of immune-associated functions within Group II (Figure 6C). The most significantly enriched biological processes included immune system process, immune response, cytokine production, inflammatory response, leukocyte migration, cell activation, regulation of cytokine production, and regulation of cellular responses to external stimuli. Cellular Component analysis identified significant enrichment of cell surface structures, plasma membrane signaling receptor complexes, integrin complexes, and membrane-associated protein complexes. Molecular function analysis revealed enrichment of enzyme binding, kinase binding, protein kinase binding, kinase regulator activity, phosphatidylinositol 3-kinase regulator activity, and cytokine-receptor activity.

Network analyses of BP, CC, and MF categories demonstrated highly interconnected functional modules associated with immune activation, leukocyte trafficking, membrane-associated signaling platforms, and intracellular kinase-mediated signaling pathways (Figure 6D).

### 3.7. Functional Characterization of Group III Genes Reveals Selective Suppression of Immune, Cytokine, and Lipid-Regulatory Programs Following VC Treatment

To further characterize conserved TAM-associated genes that were significantly downregulated following VC treatment, the 16 genes classified within Group III (Log2FC < −1) were subjected to KEGG and GO enrichment analyses. Unlike Group II, which represented transcriptionally stable genes unaffected by VC, Group III represented a smaller subset of conserved TAM-associated genes exhibiting measurable sensitivity to baculovirus exposure.

**Figure 7:**
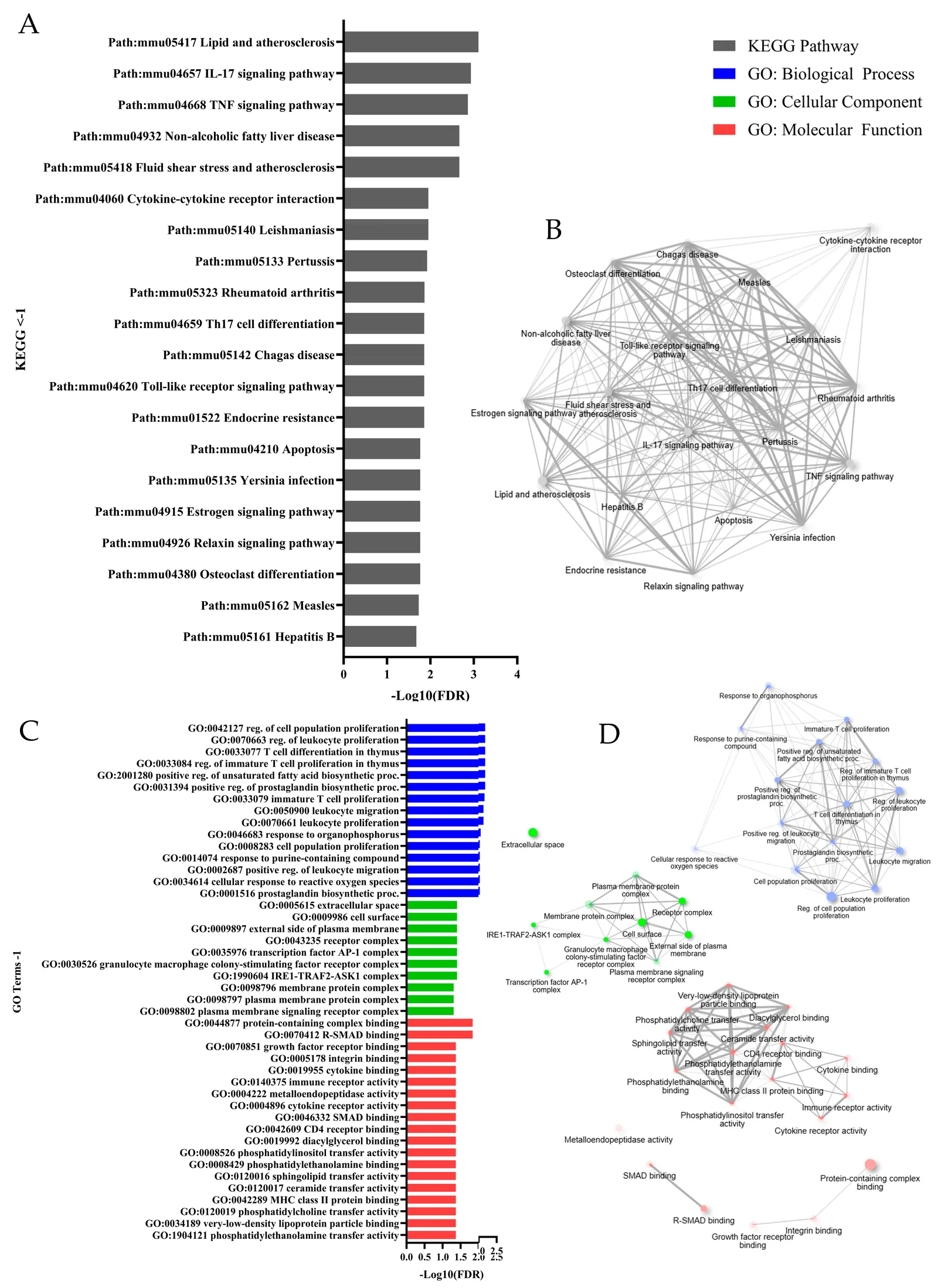
(**A**) KEGG pathway-enrichment analysis of Group III genes (Log2FC < −1) identified from the ivTAMs + VC vs. ivTAMs comparison. Group III represents conserved TAM-associated genes that were significantly downregulated following VC treatment. Enriched pathways included lipid and Atherosclerosis, IL-17 signaling pathway, TNF signaling pathway, Non-Alcoholic Fatty Liver Disease, Fluid Shear Stress and Atherosclerosis, cytokine–cytokine receptor interaction, Toll-like receptor signaling pathways, and Th17 Cell Differentiation. Statistical significance is presented as −Log10(FDR). (**B**) Protein–protein interaction (PPI) network of significantly enriched KEGG pathways identified in Group III genes. Node size corresponds to pathway connectivity, whereas edge thickness reflects the degree of shared genes between pathways. The network highlights extensive interactions among inflammatory, cytokine-mediated, metabolic, and immune-regulatory signaling pathways affected by VC treatment. (**C**) Gene Ontology (GO) enrichment analysis of Group III genes. Biological Process (BP) terms are shown in blue, Cellular Component (CC) terms in green, and Molecular Function (MF) terms in red. Enriched biological processes were primarily associated with leukocyte proliferation, leukocyte migration, T-cell differentiation, prostaglandin biosynthesis, and regulation of cell population proliferation. Cellular Component enrichment highlighted membrane-associated receptor complexes, while molecular function enrichment emphasized cytokine binding, immune receptor activity, phospholipid transfer activity, and protein complex interactions. Statistical significance is represented as −Log10(FDR). (**D**) Protein–protein interaction (PPI) networks of enriched Gene Ontology (GO) terms identified in Group III genes. The networks illustrate the functional relationships among significantly enriched GO Biological Process (BP), Cellular Component (CC), and Molecular Function (MF) categories. The BP network revealed coordinated associations among leukocyte proliferation, leukocyte migration, T-cell differentiation, prostaglandin biosynthesis, regulation of cell population proliferation, and oxidative stress-related processes. The CC network demonstrated clustering of membrane-associated immune structures, including cell surface receptor complexes, plasma membrane protein complexes, and granulocyte–macrophage colony-stimulating factor receptor complexes. The MF network highlighted functional interactions involving cytokine binding, immune receptor activity, phospholipid and sphingolipid transfer activities, CD4 receptor binding, MHC class II protein binding, growth factor receptor binding, and SMAD-associated signaling functions. Collectively, these interconnected networks indicate that VC-responsive downregulated genes are primarily associated with immune regulation, receptor-mediated signaling, and lipid-associated molecular activities.

KEGG analysis revealed significant enrichment of inflammatory and immune-associated pathways, including lipid and Atherosclerosis, IL-17 signaling pathway, TNF signaling pathway, cytokine–cytokine receptor interaction, Toll-like receptor signaling pathways, Th17 Cell Differentiation, Rheumatoid Arthritis, Leishmaniasis, and Osteoclast Differentiation (Figure 7A). Network analysis demonstrated extensive interconnectivity among these pathways, indicating substantial overlap in cytokine-regulated genes involved in immune activation and inflammatory responses (Figure 7B).

GO Biological Process enrichment further highlighted regulation of leukocyte proliferation, leukocyte migration, T-cell differentiation, prostaglandin biosynthesis, immune responses, inflammatory responses, and cellular responses to reactive oxygen species (Figure 7C,D). Cellular Component enrichment identified receptor complexes, plasma membrane signaling receptor complexes, membrane protein complexes, and granulocyte–macrophage colony-stimulating factor receptor complexes (Figure 7C,D). Molecular Function enrichment revealed cytokine binding, immune receptor activity, phospholipid transfer activity, MHC class II protein binding, CD4 receptor binding, growth factor receptor binding, and SMAD-associated regulatory activities (Figure 7C,D).

Collectively, these findings indicate that VC exposure selectively reduced expression of a limited subset of conserved TAM-associated genes involved in cytokine signaling, immune-cell communication, inflammatory activation, and lipid-associated immune-regulatory pathways.

## 4. Discussion

Macrophages possess remarkable transcriptional and functional plasticity, enabling them to respond dynamically to environmental stimuli and tissue-specific signals [29]. Within the tumor microenvironment, persistent exposure to tumor-derived factors promotes the development of tumor-associated macrophages (TAMs), which exhibit distinct transcriptional programs that differ substantially from those of naïve macrophages [30]. Consistent with this concept, the pathway-enrichment analysis revealed pronounced differences between RAW264.7 macrophages and ivTAMs + VC, particularly in immune- and inflammation-associated pathways including cytokine–cytokine receptor interaction, TNF signaling, Toll-like receptor signaling, NF-κB signaling, NOD-like receptor signaling, and JAK–STAT signaling. These findings support the successful establishment of an in vitro TAM-like phenotype and indicate extensive transcriptional reprogramming following exposure to a tumor-conditioned medium.

TAMs are widely recognized as major regulators of immune responses within the tumor microenvironment, where they contribute to immune suppression, tissue remodeling, angiogenesis, and tumor progression through the production of cytokines, chemokines, and immunoregulatory molecules [30]. Accordingly, many of the pathways identified in the present study are commonly associated with macrophage activation, inflammatory signaling, and immune communication. The elevated expression of genes such as *Il6*, *Tnf*, *Ptgs2*, *Ccl2*, *Ccl4*, *Nlrp3*, *Nod2*, and *Socs3* further supports the acquisition of a TAM-associated transcriptional profile following stimulation with tumor-conditioned medium.

Importantly, despite the substantial transcriptomic differences observed between RAW264.7 cells and ivTAMs + VC, pathway alignment analysis demonstrated only minimal differences between ivTAMs and ivTAMs + VC. Most immune- and inflammation-related pathways that distinguished TAM-like macrophages from naïve RAW264.7 cells showed little or no detectable enrichment changes following VC treatment. These findings suggest that the baculovirus vector control did not substantially alter the transcriptional programs established during TAM induction.

Gene-level comparisons further supported this observation. Although minor fluctuations in expression were detected for several inflammatory and immune-related genes, their overall expression patterns remained largely comparable between ivTAMs and ivTAMs + VC. Given the well-established plasticity of macrophages, even subtle environmental perturbations can induce measurable transcriptional responses [29]. However, the magnitude of changes observed in the present study was considerably smaller than those associated with TAM induction itself, indicating that VC exerted only limited transcriptional influence under the experimental conditions employed.

These findings are particularly relevant when considered alongside our previous study [15], which demonstrated macrophage repolarization following treatment with recombinant baculovirus constructs expressing PRRSV-associated antigens. Because the empty baculovirus vector produced only minimal pathway- and gene-level alterations in the current study, the macrophage repolarization observed previously is more likely attributable to antigen-specific effects rather than to the baculovirus backbone alone. These findings provide a controlled in vitro framework for distinguishing vector-associated from recombinant antigen-associated transcriptional responses. However, because the present experiments were performed using a murine RAW264.7-derived TAM-like model, the findings should not be directly extrapolated to primary macrophages or veterinary target species without additional validation. In addition, the present study used RAW264.7-derived TAM-like macrophages rather than primary macrophages from veterinary target species. Therefore, the observed transcriptional responses should be regarded as model-specific, and validation in primary macrophages, including porcine macrophages where relevant to PRRSV-associated applications, will be necessary before drawing conclusions regarding veterinary vaccine performance.

Tumor-associated macrophages exhibit highly conserved transcriptional programs across multiple tumor types and species despite substantial heterogeneity within the tumor microenvironment [30,31]. Recent transcriptomic studies have demonstrated that TAM populations share common functional characteristics, including cytokine production, inflammatory regulation, extracellular matrix remodeling, immune suppression, and promotion of tumor progression [32,33]. Therefore, comparison of experimentally generated TAM models with human TAM datasets represents an important strategy for evaluating biological relevance and translational applicability.

The identification of 135 overlapping genes, corresponding to 12.88% of the human TAM-associated gene set, indicates partial conservation of TAM-associated transcriptional signatures rather than comprehensive recapitulation of the human TAM transcriptome. Among these 135 overlapping genes, 88 exhibited less than a two-fold difference in relative expression changes between the human and murine datasets, corresponding conceptually to less than one cycle difference in PCR-based amplification. Regression analysis of these 88 genes yielded an R^2^ value of 0.4383, indicating a positive, although moderate, correspondence in their expression patterns across the two datasets. Taken together, these findings support the use of the murine ivTAM system as a simplified experimental model sharing selected transcriptional features with human TAMs, while differences arising from species, tumor context, cell source, and experimental conditions preclude interpretation of the model as a complete surrogate for human TAM biology. Although species-specific and tumor-specific differences are expected, previous transcriptomic studies have demonstrated that TAM populations from both human and experimental models share common immune-regulatory and inflammatory programs despite considerable heterogeneity within the tumor microenvironment [32,34,35]. The overlap observed in the present study therefore supports the translational relevance of the murine ivTAM model and indicates conservation of core TAM-associated biological processes across species.

The conserved expression of chemokines such as *CCL2*, *CCL3*, and *CCL4* is particularly noteworthy because these molecules contribute to leukocyte recruitment and communication within the tumor microenvironment. Similarly, inflammatory mediators including *TNF*, *IL6*, and *PTGS2* are recognized components of TAM-associated signaling networks that regulate immune responses and tumor progression [3,30]. The presence of these genes in both human and murine datasets suggests that the biological processes driving TAM polarization were successfully reproduced under the experimental conditions used in this study.

Importantly, the corresponding expression analysis demonstrated that the majority of these conserved TAM-associated genes remained relatively stable following VC treatment. Despite the intrinsic immunostimulatory potential of baculovirus reported in previous studies [12,13], the transcriptional patterns of overlapping human TAM-associated genes were largely preserved in ivTAMs + VC. These observations further support the conclusion that VC induced only limited transcriptional perturbation and was insufficient to disrupt the conserved TAM program established by a tumor-conditioned medium.

Together, the cross-species conservation observed in this study provides supportive evidence that the in vitro murine TAM model shares conserved transcriptional features with published human breast cancer TAM datasets. In addition, the limited transcriptomic alterations observed following baculovirus vector control treatment suggest that the core TAM-associated transcriptional identity was largely preserved under the experimental conditions evaluated. These findings provide a useful framework for interpreting subsequent recombinant baculovirus-mediated responses, suggesting that transcriptional changes observed following A1 stimulation are more likely attributable to antigen-specific activity than to the baculovirus vector backbone itself.

Macrophage activation is now understood as a broad transcriptional spectrum rather than a fixed M1/M2 binary state, and transcriptome-based classification is widely used to capture these dynamic functional states [36,37,38]. Consistent with this concept, the present study classified the 135 conserved TAM-associated genes into three groups according to Log2FC magnitude. The predominance of genes within Group II following VC treatment indicates that the baculovirus vector control produced only limited transcriptional deviation from untreated ivTAMs, supporting the stability of the established TAM-like state.

This relatively stable expression pattern is important because macrophages are highly sensitive to environmental signals and can rapidly remodel their gene-expression programs in response to inflammatory, metabolic, or tissue-derived cues [29,39]. Although baculovirus has been reported to activate innate immune signaling, the present classification shows that most conserved TAM-associated genes remained within the −1 to +1 Log2FC range after VC exposure. Thus, under the present experimental conditions, VC did not induce broad transcriptional reprogramming of the conserved TAM gene signature. These results are in agreement with previous studies that demonstrated that baculoviral DNA can activate innate immune-signaling pathways through pattern-recognition receptors, resulting in the induction of type I interferons, inflammatory cytokines, and antiviral responses [12,13].

In contrast, A1 treatment produced a markedly different expression pattern using the same gene order and classification framework. Many genes that remained stable following VC treatment showed increased expression after A1 stimulation, indicating that A1 exerted biological activity beyond the vector backbone. This supports the interpretation that the observed A1-mediated changes are primarily antigen-dependent rather than nonspecific effects of baculovirus exposure, consistent with our previous report showing macrophage repolarization following recombinant baculovirus-expressed PRRSV antigen treatment [15].

The functional annotation in Figure 3A further shows that these conserved genes are distributed across biological processes related to signal transduction, stress response, immune system regulation, cellular localization, and proliferation. These categories are consistent with the known complexity of macrophage functional programming, in which transcriptional regulation integrates immune activation, tissue adaptation, metabolic remodeling, and environmental sensing [36,40,41]. Therefore, the limited changes observed following VC treatment suggest preservation of the conserved TAM-associated transcriptional architecture.

Cross-species macrophage studies have shown that human and mouse myeloid populations can share conserved core transcriptional programs while retaining tissue- and context-specific features [41,42,43]. In this context, the stability of the 135 conserved human–murine TAM-associated genes after VC treatment further supports the minimal transcriptomic changes in the vector control. Conversely, the broader changes induced by A1 suggest that recombinant antigen stimulation can selectively modify conserved macrophage-associated pathways that are not substantially altered by VC alone.

Taken together, these findings reinforce the role of VC as an appropriate baseline control for baculovirus-based vaccine studies. The limited transcriptional perturbation induced by VC provides essential evidence that the baculovirus backbone alone does not substantially disrupt conserved TAM-associated gene programs, whereas A1 produces a distinct transcriptional response consistent with antigen-specific macrophage modulation.

One of the primary objectives of the present study was to determine whether transcriptional responses observed following recombinant antigen stimulation could be attributed to the baculovirus vector backbone itself. If A1-induced biological effects were primarily driven by VC-associated mechanisms, genes altered by VC would be expected to exhibit similar directional responses following A1 treatment. However, linear regression analyses demonstrated extremely low coefficients of determination across all three gene categories, indicating an absence of meaningful association between VC-induced and A1-induced transcriptional responses.

The lack of correlation observed in Group I genes suggests that transcripts positively influenced by VC treatment do not predict the magnitude or direction of transcriptional responses following A1 stimulation. Likewise, genes classified within Group II, representing the largest proportion of conserved TAM-associated transcripts, remained poorly correlated between the two comparisons despite their relative stability following VC treatment. 

Macrophage activation is regulated through complex transcriptional networks involving cytokine signaling, pattern-recognition receptors, metabolic pathways, and epigenetic regulatory mechanisms that collectively determine functional polarization states [37,44,45,46]. These interconnected regulatory layers allow macrophages to generate distinct transcriptional programs in response to different environmental stimuli while maintaining core lineage-specific functions. The near-complete absence of correlation observed in the present study therefore suggests that A1 activates regulatory programs fundamentally differently from those associated with baculovirus vector exposure. Distinct inflammatory stimuli are known to induce stimulus-specific transcriptional networks that generate unique gene-expression signatures despite acting on the same immune cell population [47]. The divergence observed between VC- and A1-induced responses is therefore consistent with activation of separate downstream regulatory pathways.

Previous studies have shown that baculovirus particles can induce innate immune responses through Toll-like receptor-dependent pathways and interferon-associated signaling mechanisms [13,14]. Nevertheless, the current regression analyses indicate that these modest vector-associated effects do not account for the broader transcriptional responses induced by A1. Instead, the observed divergence supports the hypothesis that recombinant antigen-mediated signaling, rather than baculovirus-mediated stimulation, represents the primary driver of transcriptional remodeling.

Furthermore, these findings suggest that A1 selectively modulates conserved TAM-associated biological pathways independently of the limited effects associated with VC treatment. This interpretation is consistent with previous reports demonstrating that antigen-specific stimulation can reshape macrophage transcriptional programs and functional polarization states without reproducing the effects observed in vector-only controls [36,38,40]. The negligible correlations observed between VC- and A1-induced gene expression profiles (R^2^ = 0.0065 and 0.0005) further support the conclusion that the limited transcriptomic alterations associated with the baculovirus vector backbone do not reflect the transcriptional programs triggered by the recombinant antigen. Because A1 exerts its biological activity through receptor-mediated immune signaling, whereas VC-induced responses exhibited virtually no correspondence with these antigen-driven changes, the vector backbone appears to contribute only a minimal and mechanistically distinct transcriptomic footprint under the experimental conditions evaluated. These findings support the concept of transcriptomic limited noises of the baculovirus vector backbone within the context of macrophage polarization.

Taken together, the consistently low R^2^ values observed across Groups I, II, and III provide strong evidence that A1-induced transcriptional responses are biologically distinct from those associated with VC exposure. These results further support the suitability of VC as a negative control and indicate that subsequent immunomodulatory effects observed following A1 treatment are primarily attributable to antigen-specific activity rather than nonspecific baculovirus-mediated stimulation.

Functional enrichment analysis of Group I genes demonstrated that the subset of transcripts exhibiting positive expression changes was strongly associated with innate immune signaling, cytokine-mediated communication, and inflammatory-response pathways. The predominance of cytokine–cytokine receptor interaction, TNF signaling, IL-17 signaling, Toll-like receptor signaling, NOD-like receptor signaling, and NF-κB signaling suggests that Group I genes represent a conserved immune-response module characteristic of activated macrophages.

These pathways are central regulators of macrophage activation and orchestrate the production of inflammatory cytokines, chemokines, and antimicrobial mediators following pathogen recognition or inflammatory stimulation. Toll-like receptors and NOD-like receptors function as major pattern-recognition receptors that initiate innate immune responses through NF-κB-dependent transcriptional programs, resulting in the production of TNF-α, IL-1β, IL-6, and numerous inflammatory mediators [48,49]. The importance of these signaling systems in coordinating macrophage-mediated host defense has been further highlighted by the recognition that TLR pathways serve as key bridges between innate and adaptive immunity [50].

The enrichment of TNF signaling, IL-17 signaling, and cytokine-associated pathways further supports the involvement of coordinated inflammatory networks. TNF signaling regulates macrophage activation, survival, and cytokine production [51], whereas IL-17 signaling amplifies inflammatory responses by promoting chemokine production and leukocyte recruitment [52]. Consistently, the observed enrichment of cytokine activity, cytokine-receptor binding, and chemokine-receptor binding suggests extensive intercellular communication mediated through inflammatory cytokine networks. Cytokines and chemokines are recognized as central regulators of immune-cell trafficking and inflammatory responses in both infectious and tumor-associated microenvironments [53].

GO enrichment analysis revealed significant associations with cellular responses to interferon-γ, regulation of tumor necrosis factor production, interleukin-1β production, innate immune response, and immune response. Interferon-γ is a critical macrophage-activating cytokine that enhances antigen presentation and antimicrobial functions, while IL-1β represents one of the major inflammatory cytokines generated during innate immune activation. Previous studies have demonstrated that IL-1β functions as a central mediator of inflammatory responses and coordinates multiple downstream immune pathways [54], supporting the biological significance of the enriched GO terms observed in Group I.

Interestingly, enrichment of NOD-like receptor signaling, AGE-RAGE signaling, and several pathogen-associated pathways indicates activation of broader inflammatory surveillance systems. NOD-like receptors participate in sensing intracellular danger signals and contribute to inflammatory cytokine production, whereas AGE-RAGE signaling has been implicated in chronic inflammatory responses and oxidative stress. These observations are consistent with the concept of sterile inflammation, whereby innate immune cells activate inflammatory programs in response to endogenous danger-associated molecular patterns rather than direct microbial infection [55].

Importantly, although these genes were initially classified based on positive expression changes associated with VC treatment, the enrichment profile does not indicate widespread cellular dysfunction or transcriptomic disruption. Instead, the identified pathways represent canonical macrophage immune functions that are commonly observed in activated innate immune cells. The extensive pathway overlap observed in the KEGG and GO interaction networks suggests that multiple enriched categories are driven by a shared core of cytokine- and chemokine-related genes functioning across interconnected immune-signaling pathways.

Collectively, these findings demonstrate that Group I genes are predominantly associated with innate immune activation, inflammatory signaling, cytokine communication, and immune regulation. The enrichment of classical macrophage-associated pathways supports the biological relevance of the identified gene set and establishes a functional baseline for evaluating the immunomodulatory activity of recombinant antigen A1 beyond the effects observed with VC alone.

Unlike Groups I and III, which represented genes modestly influenced by VC treatment, Group II consisted of genes that remained transcriptionally stable following VC exposure. Consequently, the biological pathways identified within this group provide important insight into immune-regulatory programs that are largely independent of the baculovirus vector itself. The enrichment of numerous immune-associated pathways despite minimal VC responsiveness suggests that the fundamental TAM-associated transcriptional architecture remained intact following vector treatment.

Several of the most significantly enriched KEGG pathways, including TNF signaling, JAK–STAT signaling, Toll-like receptor signaling, NF-κB signaling, cytokine–cytokine receptor interaction, and C-type lectin receptor signaling, represent central regulatory mechanisms controlling macrophage activation, inflammatory responses, cytokine production, and immune-cell communication. These pathways collectively coordinate innate immune sensing and downstream inflammatory responses in macrophages and other myeloid populations [49,50,56].

The enrichment of leukocyte migration, cell adhesion molecules, leukocyte transendothelial migration, and integrin-associated cellular components further suggests preservation of pathways involved in immune-cell trafficking and cellular interactions. Integrin complexes and cell-surface receptor assemblies are essential regulators of macrophage migration, adhesion, and communication within inflammatory and tumor microenvironments [57,58].

Importantly, molecular function enrichment highlighted kinase binding, protein kinase binding, kinase regulator activity, and phosphatidylinositol 3-kinase (PI3K) regulator activity. These findings suggest that intracellular signaling machinery remained highly represented within Group II. The PI3K pathway serves as a central regulator of macrophage polarization, survival, metabolism, and cytokine production, while kinase-dependent signaling networks integrate extracellular inflammatory signals into coordinated transcriptional responses [59,60].

The observation that these immune-regulatory pathways were enriched among genes exhibiting minimal responsiveness to VC treatment has important implications for interpretation of A1-induced responses. Because Group II genes were not substantially altered by VC, the pathways represented within this group likely reflect biological processes available for subsequent modulation by recombinant antigen stimulation rather than transcriptional changes originating from the vector backbone itself.

Consequently, the enrichment profile of Group II provides evidence that the baculovirus vector exerts only limited influence on key TAM-associated signaling pathways. The preservation of cytokine signaling, innate immune regulation, leukocyte migration, cell adhesion, and kinase-mediated signaling networks supports the conclusion that the majority of conserved TAM-associated biological functions remain largely unaffected by VC treatment. This observation strengthens the interpretation that downstream responses observed following A1 stimulation primarily reflect antigen-specific immunomodulatory activity rather than vector-derived transcriptional interference.

Group III represents the most VC-sensitive component of the conserved TAM transcriptional signature. Although only a relatively small number of genes were classified into this category, enrichment analysis demonstrated that these genes participate in highly interconnected immune-regulatory networks governing cytokine production, leukocyte trafficking, T-cell differentiation, and inflammatory signaling.

The simultaneous enrichment of TNF signaling, IL-17 signaling, Toll-like receptor signaling, and cytokine–cytokine receptor interaction pathways suggests that VC exposure may modestly attenuate specific inflammatory communication circuits operating within TAM populations. These pathways are recognized as central regulators of macrophage activation and cytokine-mediated immune responses [56,61]. However, the relatively small number of genes contained within Group III indicates that such effects are restricted to specific immune modules rather than representing global transcriptional disruption.

Interestingly, enrichment of Th17 Cell Differentiation, Leukocyte Migration, Leukocyte Proliferation, and Cell Population Proliferation suggests that VC-sensitive genes are preferentially associated with immune-cell recruitment and immune-cell communication pathways. Previous studies have demonstrated that TAMs actively regulate leukocyte trafficking and immune-cell positioning within the tumor microenvironment through cytokine and chemokine signaling networks [30,31]. Therefore, modest suppression of these pathways following VC treatment may reflect transient modulation of inflammatory signaling rather than alteration of TAM identity itself.

The enrichment of membrane-associated receptor complexes, plasma membrane signaling receptor complexes, cytokine receptor activity, immune receptor activity, and MHC class II protein binding further indicates that VC-sensitive genes are concentrated within immune-recognition and signal-transduction processes. These observations are consistent with previous reports showing that baculovirus particles can interact with innate immune receptors and transiently activate pattern-recognition receptor pathways in mammalian immune cells [13,62].

Another notable finding was the enrichment of lipid-associated pathways, including lipid and Atherosclerosis, Non-Alcoholic Fatty Liver Disease, Fluid Shear Stress and Atherosclerosis, phospholipid transfer activity, sphingolipid transfer activity, and very-low-density lipoprotein particle binding. Lipid metabolism is increasingly recognized as a critical determinant of macrophage polarization and TAM functional states [38,63]. The appearance of these pathways suggests that VC exposure may induce minor alterations in lipid-associated immune regulation, although these changes remained restricted to a limited subset of genes.

RNA-seq provides a high-resolution, sequence-based and transcriptome-wide assessment of gene expression, enabling comprehensive evaluation of transcriptional changes across thousands of genes rather than a limited number of preselected targets. This approach provides detailed information on relative gene-expression patterns and enables coordinated transcriptional responses to be evaluated at both individual-gene and pathway levels. Accordingly, the present RNA-seq dataset provides a comprehensive characterization of transcriptional changes associated with VC and A1 treatment. Nevertheless, transcriptomic evidence alone does not establish functional macrophage repolarization, and orthogonal molecular or protein-level validation would further strengthen the biological interpretation of these findings.

Several limitations of the present study should be considered. First, although six independent experimental runs were conducted for each condition, RNA from these runs was pooled before sequencing, resulting in one composite RNA-seq library per condition. Consequently, biological dispersion, replicate-based confidence intervals, and within-group transcriptional variability could not be independently estimated from the sequencing dataset. The transcriptomic analyses, including differential-expression filtering, enrichment analysis, regression, and PCA, should therefore be interpreted primarily as descriptive and exploratory. Second, although RT-qPCR was used to confirm establishment of the ivTAM phenotype prior to sequencing, representative RNA-seq-derived transcriptional changes associated with VC and A1 were not independently validated by targeted RT-qPCR, protein-level measurements, or cytokine assays in the present study. Future studies using independently sequenced biological replicates together with orthogonal gene- and protein-level validation will be required to confirm these transcriptional observations. Therefore, the A1-associated changes observed in the present study are interpreted as transcriptional changes consistent with macrophage repolarization rather than definitive evidence of functional repolarization.

Importantly, comparison with Group II demonstrates that the majority of A1-responsive pathways—including cytokine signaling, immune regulation, receptor-mediated signaling, leukocyte activation, and cellular communication—remained transcriptionally stable following VC treatment. Consequently, although VC affected a small subset of conserved TAM-associated genes represented in Group III, these effects were substantially narrower than the broader transcriptional changes observed following A1 treatment. This observation supports the central conclusion of the present study that baculovirus vector exposure exerts only limited transcriptional influence on ivTAMs and does not account for the major immunomodulatory activities associated with recombinant antigen A1.

Collectively, these findings indicate that the baculovirus vector backbone is not completely transcriptionally inactive, as detectable changes were observed in a subset of TAM-associated genes. Nevertheless, under the experimental conditions examined, VC produced relatively limited transcriptomic alterations compared with the broader transcriptional response associated with A1 treatment. Therefore, the present findings should be interpreted as evidence of limited transcriptomic effects of VC under the conditions tested, rather than complete biological neutrality.

## 5. Conclusions

This study successfully established an in vitro tumor-associated macrophage (ivTAM) model using RAW264.7 macrophages stimulated with 4T1 tumor-conditioned medium and demonstrated that the resulting transcriptional profile recapitulates key features of both murine and human breast cancer TAMs. Comparative transcriptomic analyses revealed the activation of canonical TAM-associated pathways, including cytokine–cytokine receptor interaction, TNF signaling, IL-17 signaling, Toll-like receptor signaling, and NF-κB signaling, supporting the biological relevance of the model.

Importantly, evaluation of the baculovirus vector control (VC) demonstrated only limited transcriptional influence on ivTAMs. Although a small subset of conserved TAM-associated genes and pathways exhibited VC-responsive changes, the majority of TAM-related transcriptional programs remained unaffected. Functional classification of conserved human–murine TAM signatures further revealed that most immune, inflammatory, and cell communication pathways belonged to a transcriptionally stable group that was minimally altered by VC treatment.

In contrast, recombinant antigen A1 induced distinct transcriptional responses that were largely independent of VC-associated effects. Correlation of PCA analyses demonstrated independent relationships between VC-responsive and A1-responsive gene-expression patterns, indicating that the biological activities observed following A1 treatment were attributable to the recombinant antigen rather than the baculovirus delivery platform itself.

Collectively, these findings provide transcriptomic evidence that the Baculovirus Expression Vector System exerts minimal effects on TAM-associated gene expression and supports its suitability as a platform for recombinant antigen production and immunomodulatory studies. Furthermore, the established ivTAM model provides a valuable tool for evaluating macrophage-targeted therapeutics and vaccine candidates within the tumor microenvironment. Future studies incorporating in vivo experiments and validation in primary or biologically relevant macrophages will be necessary to further confirm and extend these findings.

## Figures and Tables

**Table 1:** The Phenotypes of ivTAM, M1 and M2 macrophages.

Markers	Chang’s ivTAMs	Shen’s ivTAMs	M1	M2
*IL-10*	✔	✔		✔
*Arg-1*	✔	✔		✔
*Caspase-6*	✔	✔		✔
*CD206*	✔	✔		✔
*TNF-α*			✔	
*IL-1β*	✔	✔	✔	
Reference	[27]	[28]	[28]	[28]

Note: ✔ indicates reported expression of the corresponding marker in the indicated macrophage phenotype.

**Table 2:** Twenty-three genes appeared in both GSEA and cross-species comparison analysis.

Conserved Genes	Log2FC	Immune Function
*CCL4*	−6.853	Leukocyte recruitment
*TNF*	−5.214	Inflammatory cytokine
*CCL2*	−4.737	Monocyte recruitment
*TNFSF14*	−4.253	T-cell activation
*MMP9*	−4.243	Matrix remodeling
*PTGS2*	−4.128	Prostaglandin synthesis
*CCR2*	−4.04	Chemokine receptor
*CD40*	−4.025	Macrophage activation
*OSM*	−3.962	Inflammatory cytokine
*DPYS*	−3.87	Pyrimidine metabolism
*ITGAM*	−3.609	Cell adhesion
*CISH*	−3.163	Cytokine regulation
*CSF1*	−3.138	Macrophage survival
*IL6*	−3.221	Inflammatory cytokine
*TLR3*	−2.87	Viral sensing
*MAPK13*	−2.803	Stress signaling
*ITGB7*	−2.672	Leukocyte trafficking
*CXCL2*	−2.492	Neutrophil recruitment
*FOS*	−2.367	Transcription factor
*SOCS3*	−2.238	Signal inhibition
*CCND2*	−1.949	Cell proliferation
*BCL3*	−1.851	NF-κB regulation
*NOD2*	−1.622	Bacterial sensing

## Data Availability

Data Availability Statement: The raw RNA-sequencing data generated in this study are openly available in the NCBI Sequence Read Archive (SRA) under BioProject accession number PRJNA1032217 (https://www.ncbi.nlm.nih.gov/bioproject/PRJNA1032217) (accessed on 13 August 2026). The publicly available transcriptomic datasets used for comparative analyses are available from the sources cited in the article. Additional processed data supporting the findings of this study are available from the corresponding author upon reasonable request.
